# Transfer of statistical learning from passive speech perception to speech production

**DOI:** 10.3758/s13423-023-02399-8

**Published:** 2023-10-26

**Authors:** Timothy K. Murphy, Nazbanou Nozari, Lori L. Holt

**Affiliations:** 1 Department of Psychology, Carnegie Mellon University, Baker Hall, Floor 3, Frew St, Pittsburgh, PA 15213 USA; 2 Center for the Neural Basis of Cognition, Pittsburgh, PA 15213 USA; 3 Department of Psychological and Brain Sciences, Indiana University, Bloomington, IN 47405 USA; 4 Department of Psychology, University of Texas at Austin, Austin, TX 78712 USA

**Keywords:** Statistical learning, Speech perception, Speech production, Phonetic cue weighting, Phonetic convergence, Auditory word repetition

## Abstract

Communicating with a speaker with a different accent can affect one’s own speech. Despite the strength of evidence for perception-production transfer in speech, the nature of transfer has remained elusive, with variable results regarding the acoustic properties that transfer between speakers and the characteristics of the speakers who exhibit transfer. The current study investigates perception-production transfer through the lens of statistical learning across passive exposure to speech. Participants experienced a short sequence of acoustically variable minimal pair (beer/pier) utterances conveying either an accent or typical American English acoustics, categorized a perceptually ambiguous test stimulus, and then repeated the test stimulus aloud. In the *canonical* condition, /b/–/p/ fundamental frequency (F0) and voice onset time (VOT) covaried according to typical English patterns. In the *reverse* condition, the F0xVOT relationship reversed to create an “accent” with speech input regularities atypical of American English. Replicating prior studies, F0 played less of a role in perceptual speech categorization in reverse compared with canonical statistical contexts. Critically, this down-weighting transferred to production, with systematic down-weighting of F0 in listeners’ own speech productions in reverse compared with canonical contexts that was robust across male and female participants. Thus, the mapping of acoustics to speech categories is rapidly adjusted by short-term statistical learning across passive listening and these adjustments transfer to influence listeners’ own speech productions.

The close interaction of speech perception and production is undeniable. Perception of *one’s own* speech influences speech production (e.g., Bohland et al., 2010; Guenther, 1994). For example, altering speech acoustics and feeding speech back to a talker with minimal delay results in rapid compensatory alterations to productions that are predictable, replicable, and well accounted for by neurobiologically plausible models of speech production (e.g., Guenther, 2016; Houde & Jordan, 1998).

Similarly, perception of *another talker’s* speech can influence production. Talkers imitate sublexical aspects of perceived speech in speech shadowing tasks (Fowler et al., 2003; Goldinger, 1998; Shockley et al., 2004) and phonetically converge to become more similar to a conversation partner (Pardo et al. 2017). However, results are variable and hard to predict. Shadowers imitate lengthened voice onset times (VOT), but not shortened VOTs (Lindsay et al., 2022; Nielsen, 2011; but see also Schertz & Paquette-Smith, 2023). Phonetic convergence occurs only for some utterances or some acoustic dimensions but not others (Pardo et al., 2013). Talkers may converge across some dimensions but diverge on others (Bourhis & Giles 1977; Earnshaw, 2021; Heath, 2015), making it difficult to predict which articulatory-phonetic dimensions will be influenced (Ostrand & Chodroff, 2021). Phonetic convergence is also variable across talkers’ sex (Pardo et al., 2017), with some studies reporting greater convergence among female participants (Namy et al., 2002), others among males (Pardo, 2006; Pardo et al., 2010), or more complicated male–female patterns of convergence (Miller et al., 2010; Pardo et al., 2017). In sum, the direction and magnitude of changes in speech production driven by perceived speech are dependent on multiple contributors (Babel, 2010; Pardo, 2006,) likely to include social and contextual factors (Bourhis & Giles, 1977; Giles et al., 1991; Pardo, 2006). This has made it challenging to characterize production–perception interactions fully.

Some have argued that a better understanding of the cognitive mechanisms linking speech perception and production will meet this challenge (Babel, 2012; Pardo et al., 2022). Here, we propose an approach that is novel in two ways: (1) *Statistical learning*. Instead of investigating phonetic convergence at the level of individual words, we manipulate the statistical relationship of two acoustic dimensions, fundamental frequency (F0) and voice onset time (VOT) and study the effect of perceptual statistical learning across these dimensions on listeners’ own speech. (2) *Subtlety and implicitness*. Acoustic manipulation of the statistical regularities of speech input is barely perceptible and devoid of socially discriminating information, since it is carried on the same voice, therefore allowing us to investigate the basic perception–production transfer without influence of additional (important, but potentially complicating) sociolinguistic factors.

Our approach builds on the well-studied role of statistical learning in speech perception. *Dimension-based statistical learning* tracks how the effectiveness of acoustic speech dimensions in signaling phonetic categories varies as a function of short-term statistical regularities in speech input (Idemaru & Holt, 2011, 2014, 2020; Idemaru & Vaughn, 2020; Lehet & Holt, 2017; Liu & Holt, 2015; Schertz et al., 2015; Schertz & Clare, 2020; Zhang & Holt, 2018; Zhang et al., 2021). This simple paradigm parametrically manipulates acoustic dimensions, for example, voice onset time (VOT) and fundamental frequency (F0), across a two-dimensional acoustic space to create speech stimuli varying across a minimal pair (*beer–pier*)*.* The paradigm selectively samples stimuli to manipulate short-term speech regularities, mimicking common communication challenges like encountering a talker with an accent that deviates from local norms. Across Exposure stimuli (Fig. 1A–B, red) the short-term input statistics either match the typical F0 × VOT correlation in English (canonical condition, e.g., with higher F0s and longer VOTs for *pier*) or introduce a subtle and barely detectable “accent” with a short-term F0 × VOT correlation opposite of that typically experienced in English (reverse condition, e.g., lower F0s with longer VOTs for *pier*).

**Fig. 1:**
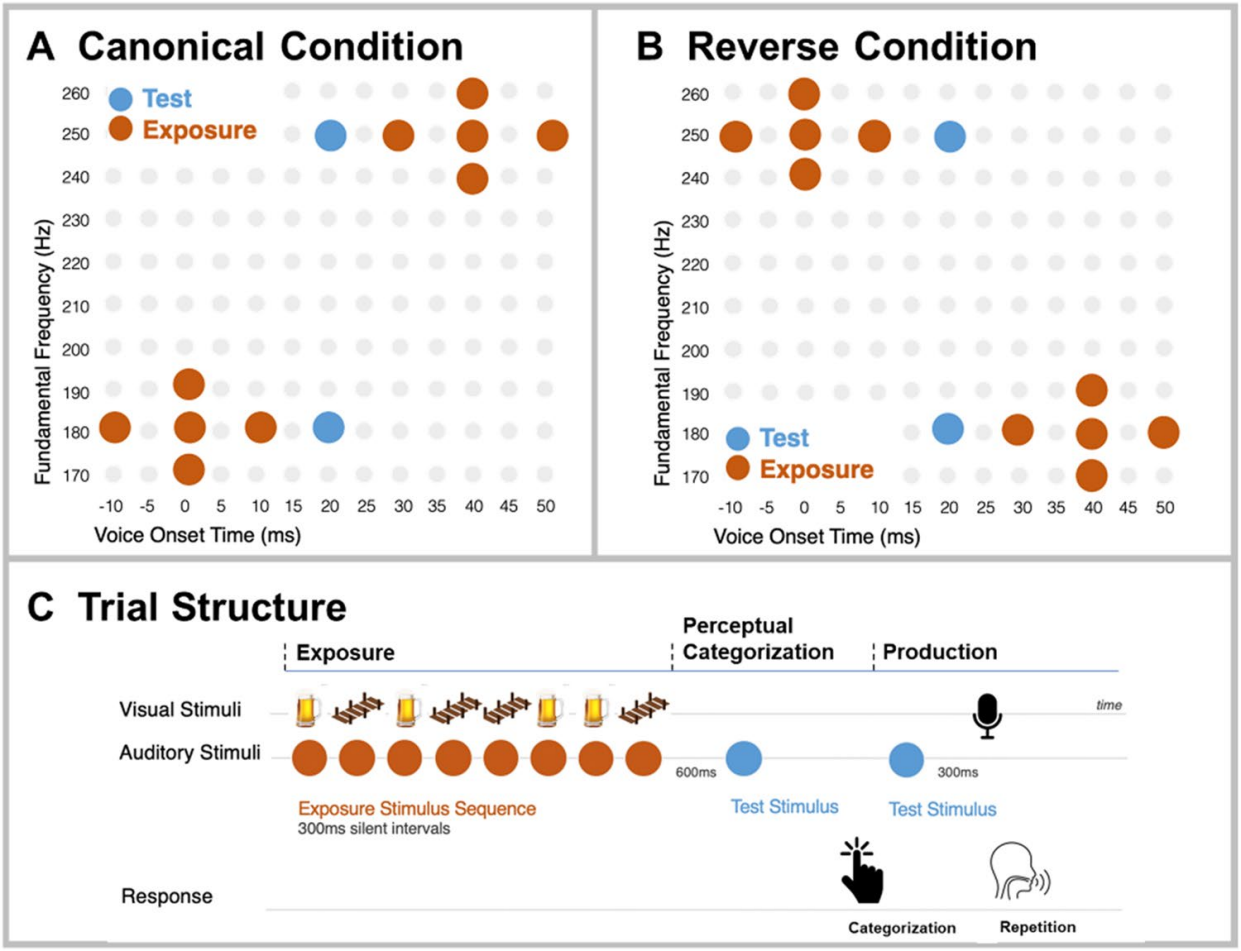
Stimulus and trial structure. **A** Canonical distribution. **B** Reverse distribution. The test stimuli (blue) have ambiguous VOT and are identical across canonical and reverse conditions. **C** Trial structure. Exposure phase: Participants listened passively to 8 exposure stimuli, each paired with a visual stimulus. Perceptual categorization phase: After 600 ms, they heard one of two test stimuli with low or high F0 and categorized it as *beer* or *pier*. Repetition phase: they heard the same test stimulus again and repeated it aloud. (Color figure online)

Test stimuli are constant across conditions (Fig. 1A–B, blue). They have a neutral, perceptually ambiguous VOT thereby removing this dominant acoustic dimension from adjudicating category identity. But F0 varies across test stimuli. Therefore, the proportion of test stimuli categorized as *beer* versus *pier* provides a metric of the extent to which F0 is perceptually weighted in categorization as a function of experienced short-term speech input regularities (Wu & Holt, 2022).

Although the manipulation of short-term input statistics is subtle and unbeknownst to the listeners, the exposure regularity rapidly shifts the perceptual weight of F0 in *beer–pier* test stimulus categorization (Idemaru & Holt, 2011). Listeners down-weight F0 reliance upon introduction of the accent. This effect is fast and robust against the well-known individual differences in perceptual weights and the variability with which individuals perceptually weight different acoustic dimensions (Kong & Edwards, 2011, 2016; Schertz et al., 2015, 2016). In all, this well-replicated finding (1) demonstrates reliable changes in the perceptual system as a function of brief exposure to subtle changes in the statistical properties of the acoustic input and (2) establishes a statistical learning paradigm as an ideal tool for examining the impact of these changes on speech production.

In the current study, we used dimension-based statistical learning to investigate whether adjustments to the perceptual space influence speech production in systematic ways. Following Hodson et al. (2023), participants passively experienced short sequences of *beer* and *pier* exposure stimuli sampling canonical or reverse distributions followed by one of the two F0-differentiated test stimuli. They categorized the test stimulus as *beer* or *pier*, then heard it again and repeated it aloud (Fig. 1C). If production is rapidly adjusted to the change in the perceptual space evoked by passive listening across statistically structured sequences of sound, we predict a down-weighting of *production* F0 in the reverse (compared with the canonical) condition. Secondarily, we examine both perception and production effects separately in male and female participants to assess whether the adjustment is influenced by participant sex.

## Methods

### Participants

Although previous studies which have used this experimental paradigm have found large effect sizes for dimension-based statistical learning in *perception*, we do not have a prior effect size for potential dimension-based statistical learning in *production*. Assuming an effect size of 0.45 with alpha of 0.05 and power of 0.8, in a within-subject design, we would need 41 participants. Because a secondary goal of this project is to assess the effect separately in male and female participants, we doubled this sample size. To allow for possible attrition, we set the target sample size of 45 male and 45 female participants.

Ninety participants (45 females) were recruited using Prolific (www.prolific.co), an online participant enrollment tool. Sex was determined by participants’ responses to the question: “What sex were you assigned at birth, such as on an original birth certificate?” In answer to a question regarding gender, 45% of participants identified as cisgender female, 48% identified as cisgender male, and 7% identified as nonbinary. Here, we used the biological variable sex.

The study was conducted under a protocol approved by the Institutional Review Board at Carnegie Mellon University. All participants were adult native-English speakers located within the United States, ages 18 to 40 years old (*M*_age_ = 28.6 years, *SD* = 6 ), and compensated at an hourly rate of $10. Following data collection, three (two female) participants were removed due to poor quality audio recordings.

### Stimuli

Acoustic stimuli were based on natural utterances of *beer* and *pier* spoken by an adult female native-English speaker digitally recorded in a sound-attenuated booth, as described by Idemaru and Holt (2020). All stimuli were derived from two initial recordings, one *beer* and one *pier*, chosen for their similarity in duration (385 ms) and F0 contour. Following the approach of McMurray and Aslin (2005), we identified 15 splice points (~2–3 ms apart, at zero crossings) in both recordings. Then, we removed the interval between *beer* onset and the first splice point and inserted a corresponding interval from the *pier*, creating a new stimulus along the VOT series. Repeating this process resulted in a fine-grained series of syllables varying in VOT from *beer* to *pier* in approximately 2–3-ms steps. From this series, syllables with VOTs of 0, 10, 20, 30, 40, and 50 ms served as stimuli. An additional stimulus with −10 ms VOT was created by taking a splice of prevoicing from *beer* and inserting it before the burst of the 0-ms VOT *beer.*

Next, we manipulated the fundamental frequency (F0) across the VOT series to create a two-dimensional F0 × VOT acoustic space, with adjustment of the F0 onset frequency (170–250 Hz in 10-Hz steps) at vowel onset manipulated manually using Praat 5.3 (Boersma & Weenink, 2021). The F0 contour decreased quadratically to 150 Hz at stimulus offset. Stimuli were normalized to the same root-mean-squared amplitude.

We sampled three types of stimuli from the F0 × VOT acoustic space. Exposure stimuli conveyed a specific F0 × VOT short-term regularity (Canonical, Reverse) across passive listening (Fig. 1C, Exposure). They possessed unambiguous VOTs diagnostic of /b/ (−10, 0, 10 ms) and /p/ (30, 40 ,50 ms) and F0 frequencies spanning 170, 180, 190, 240, 250, 260 Hz. The canonical condition stimuli (Fig. 1A, red) were sampled to exhibit the typical English F0 × VOT relationship (Abramson & Lisker, 1985), with *beer* associated with shorter VOT (−10, 0, 10 ms) and lower F0 (170, 180, 190 Hz) and *pier* associated with longer VOT (30, 40, 50 ms) and higher F0 (240, 250, 260 Hz). The reverse condition stimuli (Fig. 1B, red) reversed this F0 × VOT correlation; shorter VOTs consistent with *beer* were paired with higher F0s and longer VOTs signaling *pier* were paired with lower F0s. We constructed each trial as a sequence of four *beer* (short VOT) and four *pier* (long VOT) stimuli randomly selected from either the canonical or reverse distributions, and randomly ordered with 300-ms interstimulus silent intervals (Fig. 1C).

Test stimuli (Fig. 1, blue) served as both the probe for perceptual categorization and elicitation of speech production in the auditory repetition task. Test stimuli possessed a constant, perceptually ambiguous VOT (20 ms; see Idemaru & Holt, 2020) and either a high F0 (250 Hz) or a low F0 (180 Hz; Fig. 1A–B, blue). Two stimuli with unambiguous VOTs and high or low F0s (*beer:* 0 ms VOT, 180 Hz F0; *pier*: 40 ms VOT, 250 Hz F0). Forty-eight trials with unambiguous test stimuli were included to ensure participants did not perceive only unusual sounding probes.

### Procedure

Online participants recruited via Prolific were automatically directed to the experiment, hosted on the online experimental platform Gorilla (www.gorilla.sc, Anwyl-Irvine et al., 2018, 2021). Participants were required to use the Chrome browser, and all speech was presented in lossless FLAC format. Participants first completed consent and a simple demographics survey and then underwent a brief psychophysical check for compliance in wearing headphones using the dichotic Huggins pitch approach (Milne et al., 2021). Participants who did not pass the headphone check did not proceed to the experiment. Subsequently, a microphone check confirmed that participants’ browsers and microphones were recording speech utterances.

The experiment then commenced, expanding the perceptual protocol of Hodson et al. (2023) to examine transfer to production. Participants were instructed about the trial structure via written instructions. As illustrated in Fig. 1C, each trial had three phases: *exposure*, *perceptual categorization*, and *repetition*. In the exposure phase participants passively listened to a sequence of eight exposure stimuli (four short VOT <15 ms signaling *beer* and four long VOT >25 ms signaling *pier,* randomly ordered) separated by 300 ms of silence (5,900 ms total duration). As stimuli played diotically over headphones corresponding clipart images (*beer* for <15 ms VOT, *pier* for >25 ms VOT) appeared, synchronized to sound onset. The next phase, perceptual categorization, began with 600 ms of silence. Participants then heard a test stimulus with perceptually ambiguous VOT (20 ms) and either low (180 Hz) or high (250 Hz) F0 and categorized it as *beer* or *pier* via a keyboard response guided by on-screen text indicating the key/response correspondence as well as a question mark to indicate the need to respond. The repetition phase began immediately after response. Participants heard the same test stimulus and, 300 ms later, saw an image of a microphone that signaled them to repeat the test stimulus aloud. Participants’ utterances were recorded over their own computer microphone and stored digitally as .weba files.

The perceptual categorization and repetition phases were identical across blocks. Blocks differed in the distinctive (canonical, reverse) short-term regularities conveyed by the exposure phase. The first block was always canonical, with subsequent blocks alternating between reverse and canonical blocks. This resulted in 248 test trials (124 canonical, 124 reverse; blocks of 40–42 trials) presented across six blocks. Two of the three canonical blocks were composed of 41 trials while the third was composed of 42 trials. A small programming discrepancy led to two of the three reverse blocks having 42 trials whereas the third had 40 trials.

Among the 248 test trials, 200 trials (100 canonical, 100 reverse) presented ambiguous test stimuli to assay dimension-based statistical learning in perception and its transfer to production. The remaining 48 trials (24 canonical, 24 reverse) presented unambiguous stimuli so that participants did not perceive only unusual sounding probes. Ambiguous and unambiguous stimuli were randomized within condition (canonical, reverse). Participants had 15-s breaks after each 15 trials and between blocks*.*

### Production F0 measurements

We designed custom Praat and R scripts to extract F0 from the speech productions. In Praat (Version 6.1.51), “To TextGrid (silences)...” identified and isolated word productions in the 2.5-second audio recordings. Then, “To Pitch (ac)” characterized the F0 frequency of first 40 ms of voicing, where F0 differences between onset obstruent consonants are typically most pronounced (Hanson, 2009; Hombert et al., 1979; Lea, 1973; Xu & Xu, 2021). After F0 values were log transformed, outliers ±3 standard deviations relative to a participant’s mean F0 were removed from further analyses. Next, *z*-score normalization on a by-participant basis accounted for F0 variability across talkers that is impacted by multiple factors, including sex (Titze, 1989). Thus, a F0 value of 0 represented the mean F0 for a participant across all productions and values of ±1 corresponded to a standard deviation above and below the mean, respectively. Normalization provided a means of aligning F0 variability across participants prior to group analyses.

### Analysis

Statistical analysis involved mixed-effects models via the *lme4* package (Bates et al., 2014) in R (Version 4.1.3; R Core Development Team, 2021). In keeping with recommendations of Barr et al. (2013), we strove for including the maximal random effects in the models. Most models, however, did not tolerate the maximal random effect structure. For consistency, we report the models with random intercept of both subjects and items, which were tolerated by all models. The former captures variability among subjects; the latter among exposure sequences that changed from trial to trial. To assure that excluding random slopes did not radically alter any of the main conclusions, we also report the output of the models with the largest random effect structure tolerated by each model in Appendix 2.

For perceptual categorization data, a logit mixed-effects logistic regression model included a binary response (*beer, pier*) as the dependent variable. The model included condition (canonical, reverse), test stimulus F0 (low F0, high F0), and participant sex (male, female) and their two- and three-way interactions as fixed effects, and by-subject and by-item random intercepts included. For speech production data, a continuous *z*-score normalized F0 dependent measure allowed for a standard (non-logit) linear mixed-effects model. Here, too, fixed effects of condition, test stimulus, sex and its interactions were modeled, with by-subject and by-items modeled as random effects. Dependent categorical variables were center coded (1 vs −1). *P* values were based on Satterthwaite approximates using the *LmerTest* package (Version 3.1-3; Kuznetsova et al., 2017). Analyses collapsed data from the three canonical blocks and, separately, from the three reverse blocks.

We conducted the production analyses in two steps: (1) Our first analysis used test stimulus F0 to predict production F0. This analysis is parallel to the perceptual analysis and captures the whole process, which includes the change to perception as well as changes to production. (2) Our second analysis used perceptual responses as the main predictor of production F0. This analysis already partials out the contribution of perceptual changes as a function of exposure to the canonical and reverse distributions, which allows us to isolate the production component of transfer. The data, analysis code, and full tables of the results are available (https://osf.io/cwg4d/).

## Results

### Perceptual categorization

**Fig. 2:**
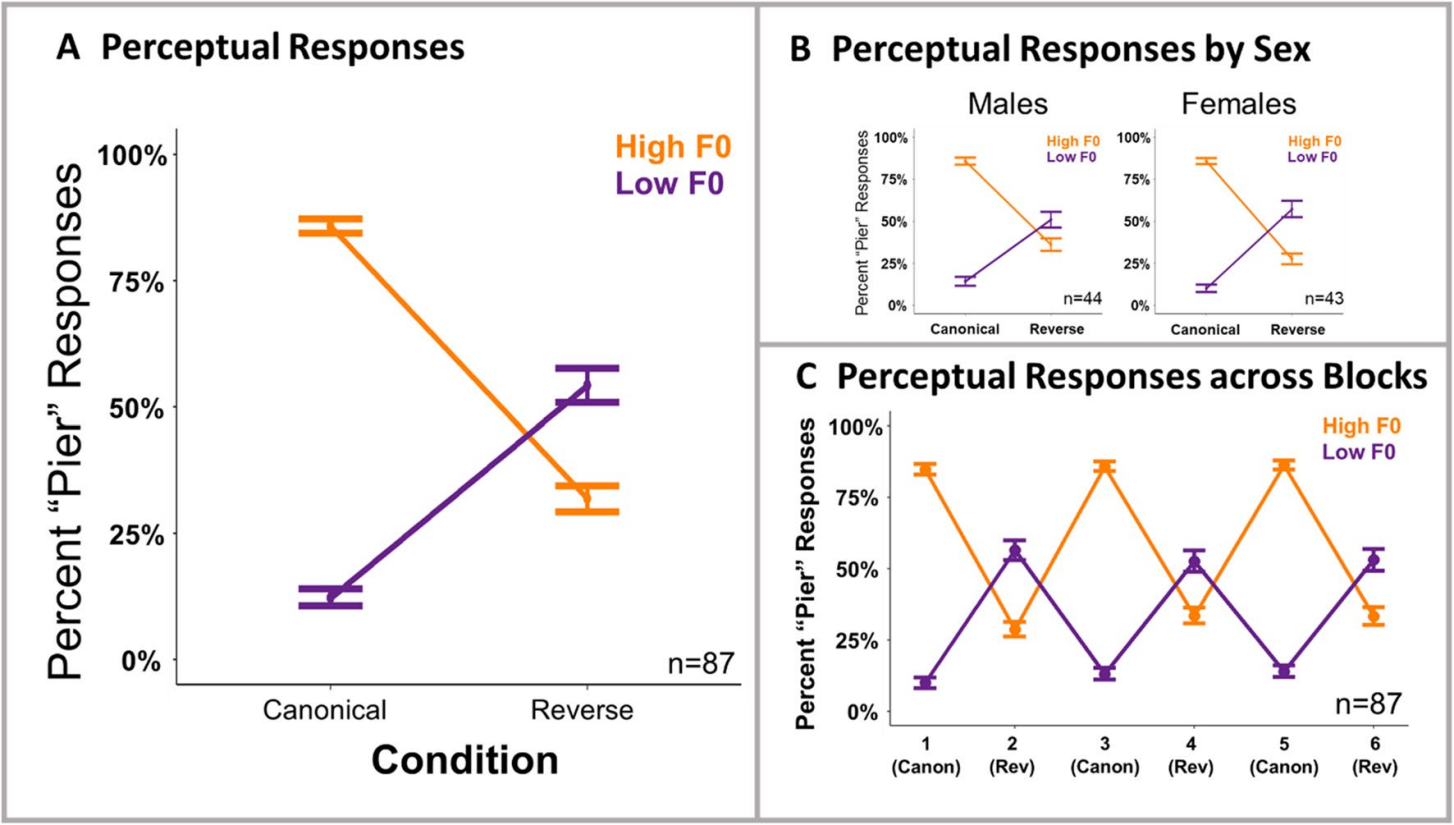
Results of perceptual categorization. Percentage of *pier* responses to high and low F0 test stimuli in canonical and reverse conditions are shown at the group level (**A**), broken down by sex (**B**), and broken down by blocks (**C**). Averages reflect subject means ± *SE*. (Color figure online)

Figure 2 plots categorization responses as a function of canonical and reverse short-term speech regularities. Table 1 presents the results of the analysis.

**Table 1:** Regression table—Perception

*Predictor*	β	*SE*	*z*	*p*
(Intercept)	−0.23	0.10	−2.26	.024
Condition	0.13	0.08	1.48	.139
Test cue F0	0.84	0.08	9.94	<.001
Sex	−0.05	0.06	−0.83	.406
Condition:Test cue F0	1.36	0.08	16.09	<.001
Condition:Sex	−0.04	0.02	−1.68	.094
Test cue F0:Sex	−0.01	0.02	−0.47	.635
Condition:Test cue F0:Sex	0.14	0.02	6.39	<.001

Reference levels are condition (reverse), target stimulus F0 (low F0), sex (male)

As expected, there was a main effect of test stimulus F0, such that the test stimulus with the high F0 was more likely to be labeled as *pier* (*z* = 9.94, *p* < .001). Crucially, as in prior studies of dimension-based statistical learning, there was a significant interaction of test stimulus F0 and condition (*z* = 16.09, *p* < .001). Passive exposure to short-term speech input regularities impacted the effectiveness of F0 in signaling *beer–pier* category identity. Neither the main effect of sex, nor its interaction with condition was significant.^1^ There was, however, a significant three-way interaction between sex, condition, and test stimulus F0 (*z* = 6.39, *p* < .001). To better understand the nature of this interaction, we conducted separate tests on male and female participants. The results showed significant Condition × Test stimulus F0 interactions for both male and female participants, with a larger coefficient for female participants (ꞵ = 1.22, *SE* = .09, *z* = 14.19, *p* < .001; ꞵ = 1.49, *SE* = .08, *z* = 17.54, *p* < .001, for males and females respectively).

In summary, listeners relied on F0 to guide decisions about speech category identity when local speech input regularities conformed to English norms. When regularities shifted to create an “accent,” F0 was much less effective in signaling the speech categories. This replicates Hodson et al. (2023), who first demonstrated that passive exposure to speech elicits dimension-based statistical learning. Adding to that result, we also showed that the effect is robust in both male and female participants. Next, we examine the influence of this perceptual statistical learning on production.

### Repetition (speech production)

**Fig. 3:**
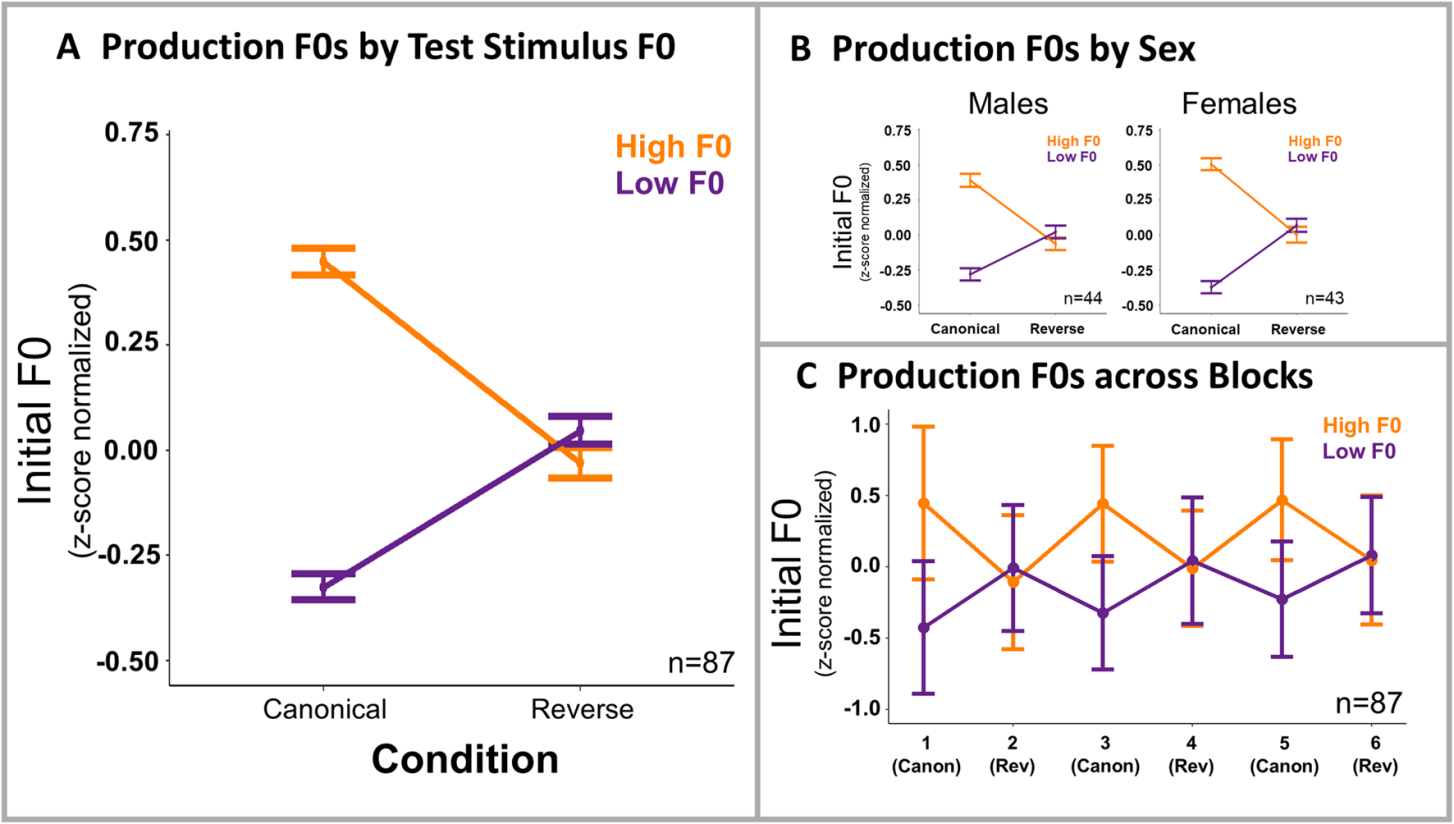
Results of repetition. F0 values in speech production by test stimulus F0 are shown at the group level (**A**), broken down by sex (**B**), and broken down by blocks (**C**). Averages reflect subject means ± *SE*. (Color figure online)

Figure 3 plots *z*-score-normalized speech production F0s elicited in response to high and low F0 test stimuli in the context of canonical and reverse short-term speech regularities. As described under Analyses, two models were run on these data. The first model predicted changes to production F0 as a function of test stimulus F0. Table 2 presents this model’s results. As in perceptual categorization, there was a significant effect of test stimulus F0, such that the high F0 test stimulus prompted a larger magnitude normalized F0 than the low F0 test stimulus (*t* = 15.36, *p* < .001). There was also a significant effect of condition, such that productions made in the canonical condition exhibited a higher F0 than the reverse condition (*t* = 2.27, *p* = .026). The interaction between test stimuli F0 and condition was significant (*t* = 19.02,* p* < .001) in a manner consistent with transfer of perceptual statistical learning to production.

**Table 2:** Regression table—Production (by test stimulus F0)

*Predictor*	β	*SE*	*t*	*p*
(Intercept)	0.01	0.01	0.50	.617
Condition	0.03	0.01	2.27	.026
Test stimulus F0	0.18	0.01	15.36	<.001
Sex	0.002	0.01	0.31	.760
Condition:Test stimulus F0	0.22	0.01	19.02	<.001
Condition:Sex	−0.01	0.01	−1.28	.199
Test stimulus F0:Sex	0.04	0.01	4.84	<.001
Condition:Test stimulus F0:Sex	0.03	0.01	3.50	<.001

Reference levels are condition (reverse), target stimulus F0 (low F0), sex (male)

There was no significant main effect of sex, though there was a significant three-way interaction among sex, condition, and test stimulus F0 (*t* = 3.50, *p* ≤ .001). Separate post hoc tests on male and female data revealed a significant interaction between condition and test stimulus F0 for both groups, with a larger coefficient for female participants (ꞵ = 0.19, *SE* = 0.02, *t* = 12.87, *p* < .001; ꞵ = 0.25, *SE* = 0.01, *t* = 20.07, *p* < .001, for males and females, respectively).

**Fig. 4:**
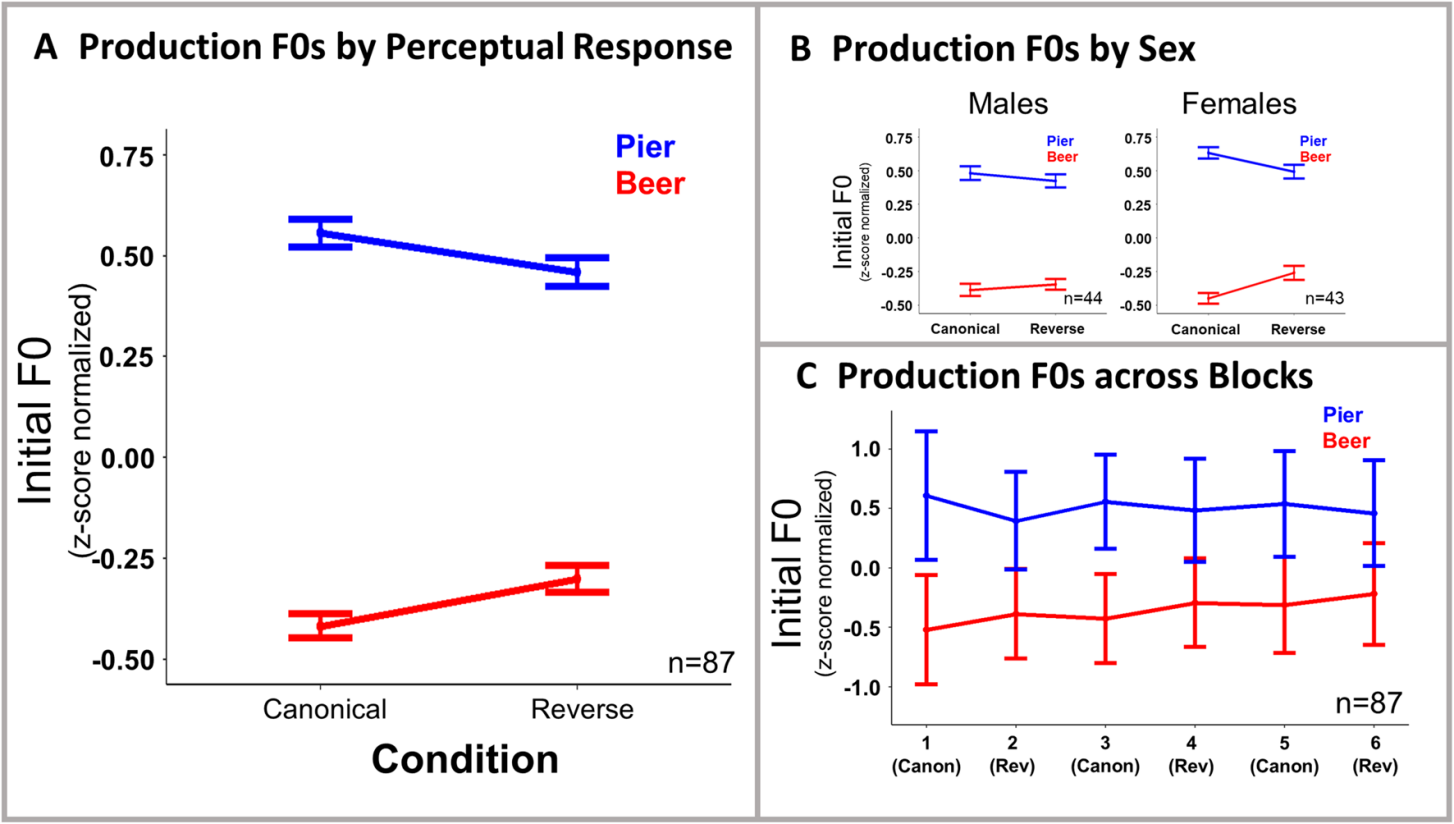
Results of repetition. F0 values in speech production by perceptual responses are shown at the group level (**A**), broken down by sex (**B**), and broken down by blocks (**C**). Averages reflect subject means ± *SE*. (Color figure online)

These results suggest that production is affected by the manipulation of short-term regularities in speech perceived passively. However, it is possible that the results are driven by changes to perception and not production. Our second analysis addresses this issue by modeling changes to production F0 as a function of participants’ perceptual choices, thus removing the variance due to the influence of test cue F0 on perception. Figure 4 shows production F0 changes based on perceptual responses, and Table 3 summarized the results of this analysis.

**Table 3:** Regression table—Production (by perceptual response)

*Predictor*	β	*SE*	*t*	*p*
(Intercept)	0.04	0.01	4.32	<.001
Condition	0.01	0.01	0.77	.443
Perceptual Response	−0.44	0.01	−58.32	<.001
Sex	0.01	0.01	0.97	.334
Condition:Perceptual Response	−0.07	0.01	−8.75	<.001
Condition:Sex	−0.005	0.01	−0.65	.513
Perceptual Response:Sex	−0.02	0.01	−3.36	.001
Condition:Perceptual Response:Sex	−0.03	0.01	−4.62	<.001

Reference levels are condition (reverse), perceptual response (pier), sex (male)

As seen in Fig. 4, when the contribution of perception is removed, the effect size clearly diminishes. The question is: Is there a significant production effect beyond those captured by perception? The results of the analysis suggest that there is. In addition to the main effect of perceptual response, there was a significant interaction between perceptual response and condition (*t* = −8.75, *p* < .001), indicating within-word changes to F0 in productions as a function of condition. A significant interaction between perceptual response and sex (ꞵ = −0.02, *SE* = .01, *t* = −3.36, *p* = .001) was evident, indicating within-word changes to F0 as a function of sex.^2^ Moreover, there was a significant three-way interaction among sex, condition, and perceptual response (*t* = −4.62, *p* < .001) with post hoc tests revealing significant effects in both sexes, with a greater magnitude in females (ꞵ = −0.04, *SE* = .01, *t* = −3.61, *p* < .001; ꞵ = −0.09, *SE* = 0.01, *t* = −8.57, *p* < .001, for males and females, respectively). This provides evidence of true transfer of perceptual statistical learning to production. This analysis shows that evidence of transfer of statistical learning to speech production is present even when the perceptual heterogeneity expected of F0-differentiated stimuli in the reverse condition is factored out. For readers interested in changes to VOT, we have reported a series of analyses including that variable in Appendix 1.

## Discussion

The findings of this study show that subtle acoustic regularities experienced in listening to a voice impact the details of our own speech. The influence of perceptual statistical learning on speech production is rapid, can result from passive listening, and impacts sublexical aspects of speech production in both male and female participants. The transfer we observe cannot be accounted for by mimicry of speech acoustics. Mimicry would predict consistent F0 patterns across conditions, since the speech tokens that elicited speech productions were constant across the experiment. Putting mimicry aside, the transfer of F0 down-weighting in an auditory repetition task can come from two sources: changes to the perception of the stimulus and/or changes to production. Comparison between the first and subsequent analyses allows us to segregate the contribution of each source. Hypothetically the down-weighting of F0 differences in productions in the reverse condition might have arisen solely from perception, without transfer to speech production. If participants were to utter *beer* and *pier* with English-consistent F0 each time they heard a high-F0 or low-F0 target then the overall F0 difference in the reverse condition might be diminished relative to the canonical condition simply because *perceptual* down-weighting leads to greater inhomogeneity in the proportion of *beer* versus *pier* percepts in the reverse, compared with the canonical, condition. This inhomogeneity would mean that high- and low-F0 targets elicit a mix of high and low F0 productions entirely due to perception, without any transfer of learning to production.

Our first analysis, conditioned on test stimulus F0, shows the combined perception plus production effect of transfer to be of a large effect size. A second analysis conditioning production F0 according to *beer* versus *pier* categorization instead of test stimulus acoustics removes the contribution of perception and shows smaller, albeit persistent, F0 down-weighting in reverse condition productions. Together, the analyses suggest that although there is a sizable perceptual contribution, there is also a unique contribution of transfer of the effects of statistical learning to production. The fact that this influence differs in magnitude across analyses conditioned on perceptual categorization versus input acoustics also makes an important point: The long-term norms of speech production are not overwritten by the more subtle influences of rapid statistical learning evident across short-term input. This is consistent with the demonstration that auditory repetition of familiar words is largely lexical (Dell et al., 2013; Nozari & Dell, 2013; Nozari et al., 2010).

These findings align with positive reports of phonetic convergence on F0 in shadowing tasks (Garnier et al., 2013; Mantell & Pfordresher, 2013; Postma-Nilsenová & Postma, 2013; Sato et al., 2013; Wisniewski et al., 2013). At the same time, they also illustrate how our statistical learning approach can provide a solution to the challenges of capturing and characterizing phonetic convergence. One advantage is dimension selection. A priori predictions about the dimensions expected to exhibit phonetic convergence have proven challenging in the phonetic convergence literature, as beautifully demonstrated by an exhaustive search across more than 300 acoustic-phonetic features (Ostrand & Chodroff, 2021). Our statistical learning approach provides a priori predictions of the dimension impacted by convergence (Wu & Holt, 2022), eliminating the need to selectively—or exhaustively—sample dimensions across which to examine the nature of transfer.

A second advantage is the ability to make directional predictions. Dimension-based statistical learning elicits predictable, directional effects on perception. When short-term speech input provides robust information (here, VOT) to indicate category identity, secondary dimensions that depart from long-term norms of these categories (as, here, for F0 in reverse condition) are down-weighted in their influence on perceptual categorization (Wu & Holt, 2022). This has proven to be the case across consonants (Idemaru & Holt, 2011), vowels (Liu & Holt, 2015), and also prosodic emphasis (Jasmin et al., 2023) categories. This is important in that it emphasizes that the transfer to production is not simply convergence in the sense of imitation. Rather, directional sublexical adjustments in the perceptual system are carried over to the production system. As a result, we would not expect all changes to the acoustics of speech to transfer to production (see, e.g., our VOT analysis). This, in turn, may help to explain why phonetic convergence studies often yield inconsistent reports.

A third advantage is the ability to set aside sociolinguistic factors. Our manipulation of acoustic F0 was barely perceptible, and devoid of socially discriminating information because the voice was constant across conditions. With this approach, we observed transfer in both male and female participants. The consistency of our findings across sex may have been supported by our approach, which allowed us to eliminate sociolinguistic factors that may contribute to the variability of findings reported in the phonetic convergence literature (Pardo et al., 2017). A sizeable literature now exists detailing social and contextual factors eliciting convergence, such as talker attractiveness (Babel, 2012), conversational topic (Walker, 2014), and even cultural primes (Hurring et al., 2022; Walker et al., 2019). Further understanding of how these factors influence convergence will benefit from an understanding of the cognitive mechanisms of transfer (Pardo et al., 2022). Here, we put forward one such an account, in the framework of statistical learning wherein several computational approaches to the perceptual effects have been proffered (Harmon et al., 2019; Kleinschmidt & Jaeger, 2015; Liu & Holt, 2015; Wu, 2020).

At the broadest level, the results demonstrate that subtle statistical regularities experienced in passive listening to *another talker’s speech* can transfer to influence one’s own speech production. Statistical learning involving short-term regularities in perceived speech impacts sublexical aspects of speech production in a predictable manner, even when the speech targets that elicit production are held constant to prevent mimicry. In sum, by yielding specific a priori predictions of the sublexical aspects of speech expected to be impacted by transfer of statistical learning, dimension-based statistical learning across passive exposure to speech provides a valuable new framework for understanding perception-production transfer.

## Figures and Tables

**Fig. 5:**
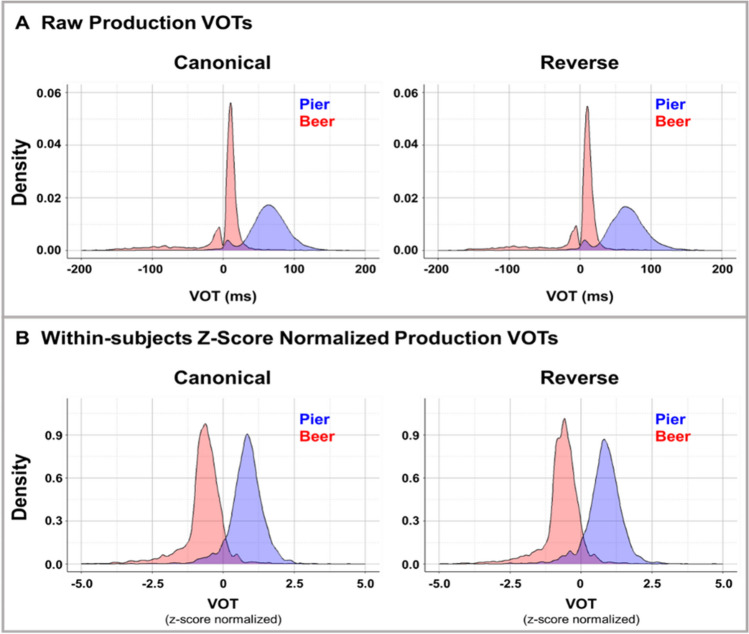
Distribution of raw production VOTs (**A**) and *z*-scored VOTs (**B**) for *beer* and *pier* in canonical and reverse conditions. (Color figure online)

## Data Availability

Data, R scripts used for statistical analyses, and results are available (https://osf.io/cwg4d/).

## Appendix 1

### VOT analysis

We measured production VOT on each trial using the Deep and Robust VOT annotator (Dr.VOT; Shrem et al., 2019), with postprocessing manual inspection. Next, we *z*-scored the VOTs following the by-participant approach described for F0.

We used VOT to verify that perceptual categorization (*beer*, *pier*) responses were followed by speech productions that corresponded to the perceptual response (e.g., longer VOTs following *pier* vs. *beer* responses). Figure 5 shows the raw (1A.A) and *z*-scored (1A.B) distribution of VOTs as a function of *beer–pier* perceptual responses. Perceptual responses were strongly associated with the VOT of subsequent productions (*t* = −141.94, *p* < .001) and production VOT distributions were not influenced by (canonical, reverse) condition (*t* = −1.51, *p* = .13, Table 4). Point biserial correlation between VOT and perceptual categorization reveals a strong relationship that is comparable across canonical and reverse conditions (*r* = 0.74, *p* < .001 for the canonical condition and *r* = 0.74, *p* < .001, for the reverse condition). In sum, participants’ *beer–pier* perceptual responses to test stimuli were followed by speech productions possessing VOTs that align with these perceptual categories.

**Table 4:** Regression table—Production VOTs (by perceptual response)

*Predictor*	β	*SE*	*t*	*p*
(Intercept)	0.05	0.01	4.16	<.001
Condition	−0.01	0.01	−1.51	.130
Perceptual response	−0.75	0.01	−141.94	<.001
Condition:Perceptual response	−0.002	0.01	−0.33	.743

Reference levels are condition (reverse), perceptual response (pier)

Given that VOT is well aligned with the perceptual response we next examined its utility as a continuous measure of participants’ intended utterance in testing transfer of statistical learning to the weighting of F0 in speech production. We fit a model predicting normalized speech production F0 as a function of utterance VOT (a continuous-measure proxy for intended production) and condition (canonical, reverse). Table 5 shows the predicted interaction (β = −0.02, *SE* = .01, *t* = −2.02, *p* = .043). The significant interaction replicates the transfer of statistical learning to speech production that persists when the F0 of the stimulus eliciting the utterance is factored out, and results are examined as a function of a participant’s *intended* speech production (here, assessed with VOT, assessed via perceptual response in Fig. 4).

**Table 5:** Regression table—Production F0s (by production VOTs)

*Predictor*	β	*SE*	*t*	*p*
(Intercept)	0.01	0.02	0.56	.580
Condition	0.01	0.02	0.64	.527
VOT	0.32	0.01	40.33	<.001
Condition:VOT	−0.02	0.01	−2.02	.043

Reference levels are condition (reverse). All VOT measures *z*-score normalized within participants

In sum, participants are largely consistent in producing words that correspond to their preceding perceptual choices. Transfer of statistical learning to speech production is observed in analyses utilizing VOT as a continuous measure of participants’ intended productions.

## Appendix 2

### Mixed-effects models with the largest random effect structure tolerated by each model

Mixed-effect models presented below share the same fixed-effect structure as corresponding models in the main manuscript but also include the largest random effect structure tolerated by each model (Table 7).

**Table 6:** Perceptual categorization

*Perceptual Response ~ Condition * Test cue F0 * Sex + (1 + Test cue F0 | Subject) + (1 | Item)*				
Predictor	β	*SE*	*z*	*p*
(Intercept)	−0.38	0.13	−2.97	.003
Condition	−0.004	0.10	−0.04	.966
Test cue F0	1.12	0.17	6.45	<.001
Sex	−0.08	0.08	−0.99	.322
Condition:Test cue F0	1.70	0.10	16.40	<.001
Condition:Sex	−0.07	0.03	−2.74	.006
Test cue F0:Sex	−0.04	0.14	−0.29	.773
Condition:Test cue F0:Sex	0.16	0.03	6.13	<.001

Reference levels are condition (reverse), test cue F0 (low F0), sex (male)

**Table 7:** Repetition (speech production by target cue F0)

Random effect structure presented in Table 2 is already the largest random effect structure tolerated by this model.	

**Table 8:** Repetition (speech production by perceptual response)

*Z-scored Production F0 ~ Condition * Perceptual Response * Sex + (1 + Perceptual Response + Condition | Subject) + (1 | Item)*				
Predictor	β	*SE*	*t*	*p*
(Intercept)	0.04	0.01	3.51	.001
Condition	0.004	0.01	0.35	.723
Perceptual Response	−0.42	0.03	−16.21	<.001
Sex	0.01	0.01	0.66	.512
Condition:Perceptual Response	−0.06	0.01	−7.33	<.001
Condition:Sex	−0.001	0.01	−0.13	.898
Perceptual Response:Sex	−0.02	0.03	−0.95	.346
Condition:Perceptual Response:Sex	−0.03	0.01	−4.64	<.001

Reference levels are condition (reverse), perceptual response (low F0), sex (male)

## Footnotes

1 The interaction between sex and condition was significant in a mixed-effects model with a random slope of test cue F0 by subject (see Table 6); however, post hoc analyses run separately in males and females revealed no significant effect of condition in either group (*z* = 0.86, *p* = .391, *z* = 0.29, *p* = .769, for males and females, respectively).

2 The interaction between perceptual response and sex was not significant in a mixed effects that included random slopes for both condition and perceptual response by subject (Table 8).
